# Heavy Enzymes and the Rational Redesign of Protein Catalysts

**DOI:** 10.1002/cbic.201900134

**Published:** 2019-07-24

**Authors:** Alan F. Scott, Louis Y.‐P. Luk, Iñaki Tuñón, Vicent Moliner, Rudolf K. Allemann

**Affiliations:** ^1^ School of Chemistry Cardiff University Main Building, Park Place Cardiff CF10 3AT UK; ^2^ Departament de Química Física Universitat de Valencia 46100 Burjassot Spain; ^3^ Department of Physical and Analytical Chemistry Universitat Jaume I Avenida de Vicent Sos Baynat, s/n 12071 Castellon Spain

**Keywords:** alcohol dehydrogenases, dihydrofolate reductases, enzyme engineering, isotope effects, molecular dynamics

## Abstract

An unsolved mystery in biology concerns the link between enzyme catalysis and protein motions. Comparison between isotopically labelled “heavy” dihydrofolate reductases and their natural‐abundance counterparts has suggested that the coupling of protein motions to the chemistry of the catalysed reaction is minimised in the case of hydride transfer. In alcohol dehydrogenases, unnatural, bulky substrates that induce additional electrostatic rearrangements of the active site enhance coupled motions. This finding could provide a new route to engineering enzymes with altered substrate specificity, because amino acid residues responsible for dynamic coupling with a given substrate present as hotspots for mutagenesis. Detailed understanding of the biophysics of enzyme catalysis based on insights gained from analysis of “heavy” enzymes might eventually allow routine engineering of enzymes to catalyse reactions of choice.

## Graphical abstract

**Protein motions in enzyme catalysis**: Studies on isotopically labelled “heavy” enzymes reveal that it is desirable to minimise the coupling of protein dynamics to catalysis. A potential roadmap for future research is proposed, with the end goal of being able to redesign protein catalysts with optimal electrostatic preorganisation for designer reactions.

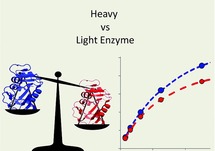

## Introduction

Enzymes are naturally occurring protein catalysts that control the chemistry of life.^1^ Their catalytic power is exploited by industry for, inter alia, waste management and the production of food, pharmaceuticals, textiles and fine chemicals.^2^ Because they are able to catalyse difficult synthetic reactions without the need for extreme temperature, high pressure or toxic chemicals and require simplified downstream processing (due to fewer unwanted side products), enzymes are increasingly becoming viewed as sustainable alternatives to synthetic catalysts, offering reductions in energy costs and in environmental damage.^2^, ^3^

However, the selectivity of enzymes is often for substrates that are not optimal for the needs of industry, a major limitation that holds back their widespread use.^4^ This problem can be addressed by the redesign of natural enzymes, either through rational design or through directed evolution. Though some key achievements have been seen, redesigning enzymes is not a simple task.4c, 5 A recent review of enzyme engineering showed that only ≈5 % of modified enzymes in a literature sample (60 enzymes produced by directed evolution and 15 computationally designed/redesigned enzymes) gave more than 10^4^‐fold increases in catalytic efficiency (*k*_cat_/*K*_M_) over the native proteins.4c To understand the cause of this failure and to become better engineers of enzymes, it is essential further to improve our understanding of the principles by which natural enzymes operate.

In a conventional understanding of enzymology, the catalytic power of enzymes comes from their ability to stabilise transition states through binding interactions, thus lowering reaction activation energies.^6^ However, antibodies that possess binding sites that are complementary to transition states either fail to catalyse reactions or display significantly lower efficiency than the natural enzymes.^7^ There are many possible explanations for this failure, including poor design of the hapten.^7^ It has also been noted that antibodies lack the residues required in order to participate in catalysis: for acid–base proton shuffling, for example.7b Also, unlike most enzymes, catalytic antibodies lack the ability to mediate conformational changes for the binding and release of substrates and products.^7^ To explain these observations, controversial enzymology models have been developed. Notably, it has been suggested that the catalytic power of enzymes is mediated through dynamic motions in the protein.^8^ This has been a topic of intense debate, and some researchers have disputed the need to invoke such “promoting motions” and have proposed that transition state theory alone can explain the catalytic power of enzymes.6b, 6e, 9

## Heavy Enzymes

The observation that isotopically labelled enzymes sometimes show reduced rates of catalysis was first made in 1969.^10^ In 2011, Schramm and co‐workers pioneered the utilisation of such isotopically labelled enzymes to probe the possible roles of protein motions in catalysis.^11^ ln this method, all nonexchangeable carbon, nitrogen and hydrogen atoms in an enzyme are replaced with their heavy counterparts—^15^N, ^13^C and ^2^H—to generate a modification with an increased mass and slower motions. The increased atomic mass alters the vibrational frequencies but according to the Born–Oppenheimer approximation leaves the potential energy surface (PES) unaltered. The rate of the chemical step is measured, and the ratio of the rate constants for the light enzyme to those for its heavy counterpart gives an enzyme kinetic isotopic effect (KIE). If a significant fraction of protein atoms has been isotopically substituted, an enzyme KIE of unity implies no significant coupling of dynamics to the chemical step, whereas a KIE above or below unity is taken to imply significant coupling of protein motions to catalysis.

**Figure 1:**
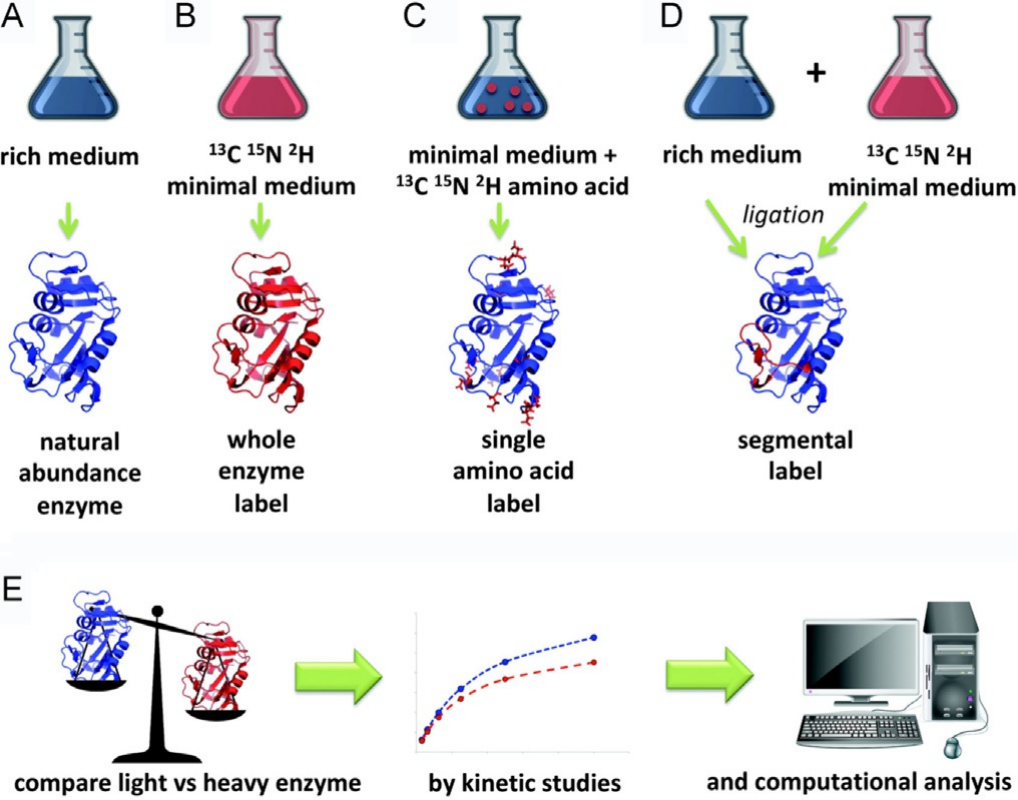
Strategies for the production and analysis of heavy enzymes. A) to D) show A) the production of natural‐abundance enzyme, B) whole‐enzyme isotope labelling, C) single‐residue isotope labelling, and D) segmental isotope labelling by production of two peptides, only one of which is labelled with heavy isotopes, that are ligated together and refolded. E) How heavy enzymes are analysed by kinetics and computational analysis.

Strategies previously employed in the literature by various groups include isotopically labelling the entire enzyme11b, 12 or labelling either of single amino acid residues^13^ or of particular segments, such as mobile loops (Figure 1).^14^ The effects of protein isotope labelling on transition states have been characterised in different enzymes including purine nucleotide phosphorylase (PNP),11a, 13, 15 HIV protease (HIV‐1 PR),11b alanine racemase,12c dihydrofolate reductase (DHFR),12b, 12h, 12j, 14a, 16 pentaerythritol tetranitrate reductase (PETNR),12d, 12e formate dehydrogenase (FDH),12f lactate dehydrogenase (LDH)12g and alcohol dehydrogenase.12k In most of these cases, isotope labelling reduced the rate of the chemical step.^11^, 12c–12e, 12g–12j, 13a, 14a, 16a These observations are often interpreted to demonstrate that protein motions couple to active‐site chemistry by engaging a probabilistic search for conformations that promote the crossing of the energy barrier.^11^, 12c–12e, 13 Nevertheless, for most of these enzymes, mechanistic studies in greater depth are needed, because computational analysis, mutagenesis studies and comparisons between homologues are often lacking.

## Dihydrofolate Reductase

**Scheme 1:**
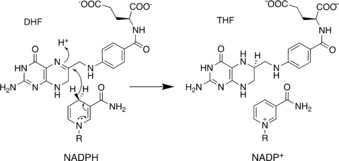
Reaction catalysed by dihydrofolate reductase.

Intensive studies on enzyme KIEs have been performed on dihydrofolate reductase (DHFR), including combined experimental and computational approaches to investigate different homologues and variants.12h, 12j, 14a, 16 DHFR catalyses hydride transfer from NADPH to tetrahydrofolate (THF, Scheme 1).^17^

**Figure 2:**
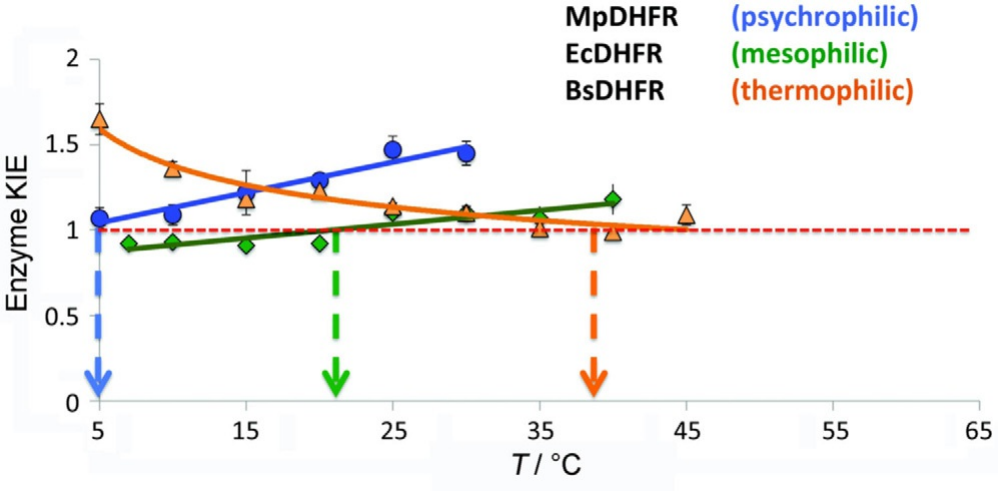
Temperature dependent pre‐steady‐state KIEs for different DHFRs at pH 7.0. Data show MpDHFR (•),12i EcDHFR (⧫)12j and BsDHFR (▴).12h The arrows indicate the temperature at which the KIE tends to unity for psychrophilic MpDHFR, mesophilic EcDHFR and thermophilic BsDHFR.

Because of the small mass of hydrogen, this reaction has a significant tunnelling component and is therefore particularly sensitive to changes in protein dynamics. The temperature dependence of the heavy enzyme KIE on hydride transfer has been investigated, by using pre‐steady state kinetics, for DHFRs from organisms that have adapted to live at different temperatures.12h–12j, 16b At physiological pH, DHFR from *Escherichia coli* (EcDHFR) shows an enzyme KIE on the hydride transfer rate of 0.93; it rises to 1.18 as the temperature is increased from 10 to 40 °C.12j The DHFR from the psychrophilic *Moritella profunda* (MpDHFR) shows an enzyme KIE that rises from 1.07 at 5 °C to 1.45 at 30 °C.12i The DHFR from the thermophile *Geobacillus* (formerly *Bacillus*) *stearothermophilus* shows the reverse trend, with the KIE falling from 1.65 at 5 °C to 1.09 at 45 °C.12h In each case, the KIE approaches unity close to the physiological temperature of the host organism; this thus strongly suggests that dynamic coupling might be at a minimum around physiological temperature (Figure 2).12h–12j, 16b Computational studies illustrate that the observed KIEs in DHFR are not due to hindered “promoting motions” but rather to increased recrossing of the transition state surface.12h–12j, 14a The increased mass has reduced the frequencies associated with protein motions, leading to a possible delay in the reorganisation of the active site environment in response to the fast changes taking place in the chemical system during barrier crossing. Incomplete environmental relaxation can induce a barrier recrossing event in which the chemical system is unable to progress to the product state and has to return towards the substrate state. Increased recrossing events can also be interpreted as a consequence of an effective “friction” acting on the reaction coordinate, as discussed later.

This hypothesis was supported by the heavy enzyme study of the conformationally restricted EcDHFR mutant N23PP/S148A, which shows an inefficient process of electrostatic preorganisation and an increase in fast‐timescale dynamics in the active site.16a These findings are consistent with those made in computational analysis of the unrelated enzymes HIV‐1 PR and PNP.^18^

To understand the precise origin of these enzyme KIEs further, it is necessary to identify the residues and regions of the protein responsible for the observed effects. In the case of EcDHFR, native chemical ligation was used to construct hybrid isotopomers in which either the N terminus—including the flexible M20 loop—was labelled and the remainder of the protein was left with natural‐abundance isotopes, or vice versa.14a Labelling of the M20 loop impacted the steady‐state kinetics, in which physical steps are rate‐limiting, but an enzyme KIE of unity was observed under pre‐steady state conditions, under which hydride transfer is rate‐limiting. Labelling the whole protein with the exception of the M20 loop restored the full enzyme KIE observed in the case of the fully labelled enzyme. This demonstrates that the origin of the enzyme KIE is not in the first 28 residues of the protein. To discover the microscopic origin of dynamic effects, it will be necessary to extend the work to label the FG and GH loops selectively.

These studies on DHFR have progressed our knowledge of protein dynamics and suggest that the catalytic rate reductions observed in heavy enzymes under non‐physiological conditions should not be taken as evidence that protein motions drive the chemical step (i.e., promoting motions).12h–12j, 16, 19 Instead, heavy enzymes present more trajectories that recross the transition state dividing surface, because the ability of the heavy protein to adapt to changes in the chemical system is less than that of the light enzyme. In other words, the isotopic substitution reduced protein motions, giving greater friction between the protein and the chemical system as it advanced along the reaction coordinate. The friction concept is a convenient way to express the effect of enzymatic degrees of freedom on a given reaction coordinate, by viewing it as an effective friction acting against the advancing of the system past the coordinate. In our treatment, for enzymes acting on their natural substrates under physiological conditions, this effect can be seen as a small perturbation of the equilibrium description assumed in transition state theory.^20^ In nature, an enzyme's active site is preorganised to work with specific substrates at a given temperature. Thus, under these conditions, barrier crossing essentially involves the degrees of freedom of the chemical system and, for the purposes of calculating activation free energies and rate constants, protein motions can be considered in equilibrium with them. Slow motions precede fast motions on the way to the transition state. The active site reorganisation needed to accommodate the charge distribution of the chemical step takes place on a different timescale from the transition state crossing. Hence, protein motions will have their greatest impact before or after the chemical step.16a, 20

This viewpoint conflicts with theories developed from work on other, unrelated enzymes.^11^, 12c–12g, 13a According to these authors, enzymatic degrees of freedom would be an integrative part of the barrier‐crossing event and are therefore important to determine the properties of the transition state. It has furthermore been suggested that DHFR is unique and that conclusions from this enzyme should not be extended to other systems.^21^ In response to this criticism, the insights gained from DHFR were further tested in another system: alcohol dehydrogenase (ADH).12k

## Alcohol Dehydrogenase

**Scheme 2:**
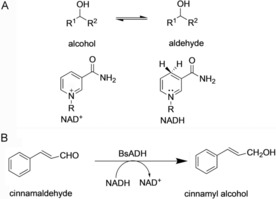
A) Reaction catalysed by BsADH. B) A biocatalytic application of BsADH to generate a valuable compound.^23^

According to the theories developed in the DHFR work, dynamic coupling is increased under non‐physiological conditions requiring greater reorganisation of the active site during the chemical step of the catalytic process. If this is correct, it therefore follows that dynamic coupling should also be increased when unnatural substrates are used, because the active site architecture is not optimised for the corresponding chemical transformations.12k To test this proposal, the promiscuous zinc‐dependent alcohol dehydrogenase from *G. stearothermophilus* (BsADH) was used.12k BsADH is a thermostable tetramer that catalyses the NAD^+^‐dependent interconversion of alcohols and aldehydes (Scheme 2).^22^ It has been used as a biocatalyst in the generation of cinnamyl alcohol, a valuable compound used for fragrance, food flavouring and synthesis of pharmaceuticals.^23^

**Figure 3:**
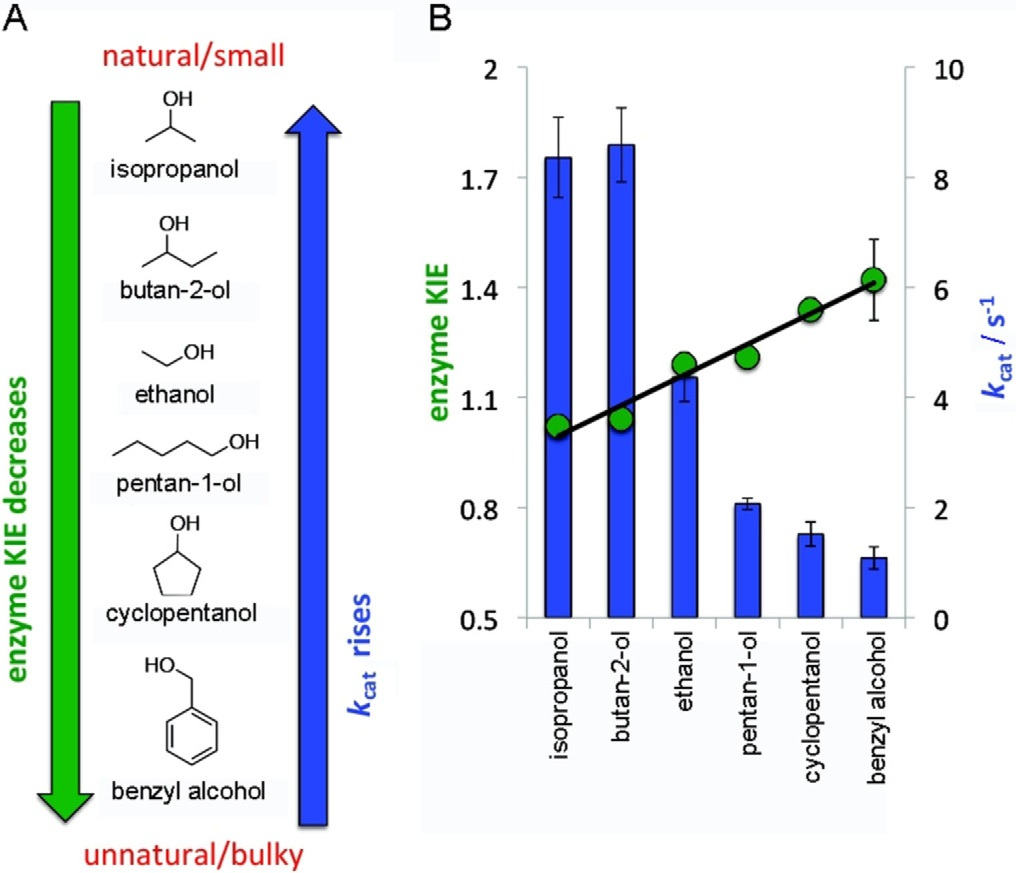
BsADH heavy enzyme KIE for a range of substrates correlated with *k*_cat_ at 20 °C. A) Structures of substrates tested. B) Enzyme KIEs are shown in circles; *k*_cat_ values are shown as bars. Data from ref. 12k.

Like DHFR, this enzyme catalyses a simple hydride transfer reaction. The heavy enzyme KIE for BsADH was measured for a range of substrates from well‐tolerated “good” substrates to poorly tolerated “bad” substrates.12k “Good” substrates were small nonconjugated molecules with values of *k*_cat_ ranging from 2 to 8 s^−1^ (Figure 3 A). “Bad” substrates were bulky and highly conjugated, with *k*_cat_ values below 2 s^−1^. Because hydride transfer is partially rate‐limiting, the heavy enzyme KIE measurements were based on *k*_cat_.^24^ No dynamic coupling was observed for any of the substrates at physiological temperature (40 °C), at which enzyme KIEs were around unity. However, at lower temperature (20 °C), the enzyme KIEs rose with inverse correlation to *k*_cat_ (Figure 3 B). The absence of dynamic coupling at physiological temperature is consistent with earlier work on DHFR and shows that dynamics do not contribute to the reaction under physiological conditions.12h–12j, 16, 19 The KIEs observed at lower temperature correlated with *k*_cat_ and confirmed the hypothesis that unnatural, bulky substrates require assistance from protein dynamics to produce a larger reorganisation of the active site.12k As a result, slightly greater protein friction is generated on the chemical system along the evolution of the reaction coordinate. This means more protein movements (femtosecond mass‐dependent protein motions) that can be coupled to the crossing of the transition state dividing surface, an event that occurs on a timescale of the same order of magnitude as the protein motions (femtoseconds).

## Future Outlook for Protein Engineering

Our growing understanding of protein dynamics could help to engineer better enzymes in the future. Theoreticians have proposed that introducing a new “promoting motion” would improve the activity of aromatic amine dehydrogenase but this has never been tested experimentally.^25^ Similarly, work on PNP has shown that the efficiency of barrier crossing in a heavy enzyme can be modified by mutations that enhance promoting vibrations.^15^ Experimentally, this resulted in the inversion of the enzyme KIE from a normal KIE of 1.31 to an inverse KIE of 0.75.^15^ However, the mutant enzyme was less catalytically efficient than the wild type. An alternative or complementary approach for engineering dynamics emerges from the study of DHFR and BsADH.12h–12k, 16, 19 In these enzymes there are no “promoting motions” but rather protein dynamics are involved in active site reorganisation under non‐physiological conditions or when poorly tolerated substrates are used. Identification and mutation of residues responsible for dynamic effects in BsADH could therefore provide a route towards the rational re‐engineering of this enzyme for unnatural substrates. It is thus now necessary to locate the region of BsADH and the particular amino acid residues responsible for dynamic effects in order to test the hypothesis that these residues are hotspots for re‐engineering of the enzyme's substrate profile. A number of experimental and computational techniques for identifying residues responsible for dynamic effects exist; they include labelling of a single amino acid (a technique previously applied to PNP)^13^ or the production of hybrid isotopomers in which one particular loop or domain of a protein is isotopically labelled.^14^ Such hybrids can be constructed by using a variety of techniques including chemical ligation, in which a peptide containing a C‐terminal thioester can be ligated to a peptide containing a free N‐terminal cysteine residue. This technique has been applied to EcDHFR.^14^ Other approaches involve the use of peptide ligases^26^ or protein *trans* splicing with split inteins.^27^ The best approach for a particular protein has to be experimentally determined and often requires time‐consuming optimisation of the ligation and subsequent refolding of the ligated chain. For the technique to become practical as a routine method for enzyme engineering, further advances need to be made in protein ligation and refolding technologies to enable easy construction of hybrid isotopomers. An alternative, complementary approach is to use computer simulations to predict the residues responsible for friction along the reaction coordinate.^18^

It may be questioned whether the gains in catalytic efficiency from such engineering will be large, given the small enzyme KIEs observed. Although the enzyme KIEs appear small, there is a significant kinetic difference between “good” and “bad” substrates. Hence, mutation of residues that hinder the progression of the reaction can translate into a measurable increase in catalytic turnover. Nevertheless, because only a small number of enzymes have to date been studied by the heavy enzyme methodology, it is currently unclear which enzymes obey the rule of minimised dynamic coupling under physiological conditions. Hence investigations of protein dynamics over a broad range of enzyme families with different chemistries and cofactors are essential.

## Summary

Heavy‐isotope labelling of proteins combined with detailed computational work is a useful tool for studying the contribution of protein motions to the catalytic step. Studies on DHFR variants have shown that dynamic effects are only significant under nonphysiological conditions that require reorganisation of the active site.12h–12j, 16, 19 This leads to the hypothesis that dynamic coupling should also be increased when unnatural substrates are used because the active site architecture is not optimised for the corresponding chemical transformations.12k By this argument, dynamic coupling indicates the extent to which an active site is suited to a particular substrate. This was confirmed through heavy enzyme studies on an alcohol dehydrogenase from *G. stearothermophilus*. Amino acid residues responsible for dynamic effects might be useful as targets for mutagenesis to create an active site optimally suited for a designed substrate. Unlike directed evolution, which requires a high‐throughput screen to assay a large number of variants, such a rational approach based on insight into dynamic effects only requires a small number of mutants to be analysed.^28^

## Conflict of interest

*The authors declare no conflict of interest*.
